# Eutirucallin, a RIP-2 Type Lectin from the Latex of *Euphorbia tirucalli* L. Presents Proinflammatory Properties

**DOI:** 10.1371/journal.pone.0088422

**Published:** 2014-02-18

**Authors:** Sanzio Silva Santana, Margareth Leitão Gennari-Cardoso, Fernanda Caroline Carvalho, Maria Cristina Roque-Barreira, André da Silva Santiago, Fátima Cerqueira Alvim, Carlos Priminho Pirovani

**Affiliations:** 1 Universidade Estadual de Santa Cruz, Centro de Biotecnologia e Genética, Ilhéus, Bahia, Brasil; 2 Medical School of Ribeirão Preto, Universidade de São Paulo, Ribeirão Preto, São Paulo, Brasil; Chinese Academy of Sciences, China

## Abstract

Lectins are carbohydrate-binding proteins that recognize and modulate physiological activities and have been used as a toll for detection and identification of biomolecules, and therapy of diseases. In this study we have isolated a lectin present in the latex of Euphorbia tirucalli, and named it Eutirucallin. The latex protein extract was subjected to ion exchange chromatography and showed two peaks with haemagglutinating activity. Polypeptides of 32 kDa protein extract strongly interacted with immobilized galactose (α-lactose > D-N-acetylgalactosamine). The Eutirucallin was obtained with a yield of 5.6% using the α-lactose column. The lectin domain has 32 kDa subunits and at least two of which are joined by disulfide bridges. The agglutinating capacity for human erythrocytes A^+^, B^+^ and O^+^ is inhibited by D-galactose. The haemagglutinating activity of Eutirucallin was independent of Ca^2+^ and maintained until the temperature of 55°C. Eutirucallin presented biological activities such as neutrophils recruitment and cytokine prodution by macrophages. The analysis of the trypsin-digested Eutirucallin by ms/ms in ESI-Q-TOFF resulted in nine peptides similar to type 2 ribosome-inactivating protein (type-2 RIP). It's partial sequence showed a similarity of 67.4 – 83.1% for the lectin domain of type-2 RIP [Ricin and Abrin (83.1%), Viscumin, Ebulin, Pulchellin, Cinnamomin, Volkensin and type-2 RIP Iris hollandica]. Our data suggest that Eutirucallin is a new member of type 2 ribosome-inactivating protein and presents biotechnological potential.

## Introduction

Species of the genus *Euphorbia* (Euphorbiaceae) usually present a series of bioactive components as an example of essential oils (eugenol), aldehydes, latex, starch, tigliane-type diterpenes (phorbol esters) and ingenane (ingenol esters), tigliane-type diterpenoids (formaldehyde), lysozymes, and lectins [1], [2], [3]. The *Euphorbia tirucalli* is origin in Africa and has acclimated to warm places of Brazil, especially in the northeast. This plant has been widely used in the practice of alternative medicine due to its wide variety of bioactive molecular constituents. Its latex is highly viscous, toxic and irritating when in contact with the skin or mucosa [2] and provide a molluscicidal activity [4], as well as larvicidal [5] and antitumor activities [6]. In addition, to the latex stimulated myelopoiesis, lead to increase of tumor resistance in mice with EAT (Ehrlich ascites tumor) [7]; and promote a negative effect of the lytic cycle activation of the Epstein-Barr virus [8]. In this work we isolated a galactose-binding lectin of *E. tirucalli* latex, Eutirucallin. We confirmed its immunostimulatory potential and presented evidence that it is a new member of the type 2 ribosome-inactivating protein.

## Materials and Methods

### Ethics statement

The animals were bred and maintained under standard conditions in the animal house of the Ribeirão Preto School of Medicine, São Paulo University, Ribeirão Preto, SP, Brazil. All animal experiments were performed in accordance with protocols approved by the Institutional Animal Care and Use Committee of the University of São Paulo (process number 082/2012).

### Extraction and analysis of latex protein

The fresh latex was obtained after small incisions in the distal branches of plants in 15 mL aliquots with distilled water in the proportion of 1∶5 (20%, v.v^−1^) and immediately kept on ice (4±2°C) [1]. The collected aqueous extract was submitted to a light homogenization for 48 h (4±2°C) and later centrifuged at 13,300 x g, 4°C for 15 min. the solubilized material was thoroughly dialyzed with distilled water (in membrane with molecular exclusion of 6 to 8 kDa; Spectra 1/Por, Spectrum) at 4°C, with changes each 8 h during 48 h. The resulting milky extract was frozen for at least 72 h; after thawing the clot was removed [31]. The resulting supernatant (almost clear) was filtered with nylon membrane (pore size 0.45 µm; Amersham Hybond, GE Healthcare) for obtaining of protein extract. The protein concentration was determined according to the Bradford method [32] on Versamax reader (Molecular Devices) with software v. 4.8 SoftMax DO_595nm_. To obtain a concentrated protein extract, it was frozen (−80°C), freeze-dried and reconstituted in phosphate buffer or tris (0.05 M) containing 0.15 M of sodium chloride (PBS and TBS, respectively). The protein extract was evaluated by SDS-PAGE (12.5%) under reducing and non-reducing denaturation conditions [33] at different heat treatments. The samples were diluted in sample buffer of SDS-PAGE with or without reducing agent (2-mercaptoethanol), incubated for 5 min at room temperature (25°C) or at 95°C. The polypeptide bands were stained with “coomassie brilliant blue G 250” (2%) and scanned in ImageScanner (Amersham Biosciences, Little Chalfont, Buckinghamshire, UK) using the software MagicScan 32 v4.6.

### Identification of the agglutinin in the protein extract

Human blood samples properly typed (A, B and O) were obtained from the Hematology Service of Ilhéus, Bahia, Brasil. The erythrocytes used in all related trials were separated from the rich plasma and buffy coat by centrifugation (2100 x g, 5 min, 25°C), and washed three times in PBS or TBS under the same conditions. Erythrocytes obtained were then resuspended in the same buffer in the concentration of 2% (v.v-1). In this assay, we have initially investigated the haemagglutinating potential of fractions obtained from the protein extract by anionic exchange chromatography. The purification procedure was developed in high performance liquid chromatography AKTATM (Amersham Biosciences). The software Unicorn 5.0 (Amersham Biosciences) was used for control and monitoring of the procedures. For each run, 1.8 mL (1.3 mg) was applied in the column (5 mL) Unicorn Mono Q-GL 5/5 (GEHealthcare Bio-Sciences, Uppsala, Sweden) previously balanced with 20 mM of Tris-HCl, pH 8.0 (balance buffer) in a loading flow of 2 mL.min-1. The protein fractions were eluted in a segmented saline gradient ranging from 0 to 0.5 M and 0.5 to 1 M (in balance buffer) with 20 vc, and spectrophotometrically monitored in A280nm. Fractions with volume of 1 mL were eluted and a 0.2 mL aliquot of each fraction was blotted onto nitrocellulose membrane (pore size of 0.45 µm; Bio-Rad, Germany) using Bio-dot SFCell system (Bio-Rad, USA), according to the manufacture's instructions. The membrane was incubated with human erythrocytes A+ in light stirring at room temperature for 30 min. The excess erythrocytes (“background”) were removed after washing under light stirring of the membrane with TBS. The positive agglutination was obtained by the observation of a cluster of red blood cells forming a blur in the support membrane. Fractions with volume of 0.2 mL were concentrated by liofilization and loaded in SDS-PAGE. The images of the agglutination in membrane and gels were digitalized in ImageScanner (Amersham Biosciences) using the software MagicScan32 v4.6. Band`s intensity from three images was analyzed by ImageMaster 2D Platinum 7.0 (GE Healthcare).

### Protein-carbohydrate-agarose binding assays

To evaluate the protein-carbohydrate interaction, 0.2 mL (200 µg) aliquots of protein extract were incubated at the same ration (1∶1, v.v-1) with agarose spheres adsorbed with different carbohydrates (Sigma Aldrich, St. Louis, USA) (*N*-acetyl-D-glucosamine, *N*-acetyl-D-galactosamine, α-lactose and D-mannose), and incubated for 120 min at 4°C. After centrifuging (400 x g, 5 min, at 4°C) the supernatant was recovered and the resin was washed thoroughly with TBS. The absorbed protein were eluted by boiling of resin (100°C, for 5 min) in 25 µL of samples buffer of SDS-PAGE with reducing agent. After centrifuging, in the sample previous conditions, the supernatant was evaluated by means of SDS-PAGE.

### Lectin purification by affinity chromatography

As stationary phase, 1.5 mL of α-lactose-agarose matrix (Sigma Aldrich, St. Louis, USA) was used, manually packed and balanced with 20 volumes of column (cv) with PBS. About 2.41 mg of protein extract was applied to the column, and the system was left to rest for 120 min at 4°C. The non-binding proteins were removed by means of washing of the matrix with balance buffer; the procedure was spectrophotometrically monitored (A280nm) until absorbance reached zero value (0 ± 0.01). The binding protein were obtained after previous incubation (2 min) with galactose (0.4 M) diluted in PBS, followed by washing of the stationary phase with the same solution in a continuous flow of 0.8 mL.min-1. The fractions corresponding to the binding and non-binding protein were collected in volumes of 0.2 and 2 mL, respectively, grouped and thoroughly dialyzed against distilled water for 48 h (with changes each 8 h) at 4°C. The eluted fractions were concentrated by diafiltration in centricon Vivaspin 6 (GE HealthCare) tube with exclusion limit of <10 kDa, for use in later assays.

### Activity and inhibition of haemagglutinating activity in microplate

Tests were developed in microtitration of 96 “U” bottom plates (Greiner Bio-one, Monroe, North Carolina, USA). The haemagglutination was determined for A+, B+ e O+ erythrocytes by means of double serial dilution (1∶2 up to 1∶128) starting from 8.4 µg (50 µL) of protein extract or 2.35 µg of lectin, and addition of an equivalent amount (50 µL) of cellular suspension at 2% in PBS. Human erythrocytes incubated with PBS were used as control. The haemagglutination was evaluated after 1 h of sedimentation at room temperature. Different monosaccharides (D-dextrose, *N*-acetyl-D-glucosamine, D-galactose and L-fucose) were used for the agglutinating activity inhibition assay in varied concentrations (50≥0.78 mmol.L-1) as potential inhibitors. The different sugars were incubated in the same proportion (v.v-1) with the lectin (0.63 µg) in room temperature for 60 min. Followed to that the suspension of human erythrocytes B+ (2%) was added. The interaction was evaluated after 60 min. the positive haemagglutination was indicated in all testes for the formation of a uniform coating of red blood cells in the surface of the U plates, and its absence was considered as negative haemagglutination. The results were presented in number of haemagglutinating units (HU, where one unit corresponds to the minimum protein concentration required to promote the complete agglutination of red blood cells) and specific haemagglutinating (SHA, corresponding to HU.mg-1).

### Influence of temperature and EDTA in the haemagglutinating activity of the lectin

The evaluation of the effect of the temperature in the lectin activity was performed in microplate, as per the previous method, after 20 min of incubation of 1.8 µg (50 µL) of lectin at different temperatures (25, 35, 45, 55, 65, 75, 85 and 95°C), and addition of cell suspension (A+) at 2% in the same proportion (v.v-1). The evaluation of Ca2+ ions dependence for the haemagglutinating activity was determined after previous incubation of lectin (1.8 µg) with 10, 50 and 100 mmol.L-1 of EDTA chelating agent for 18 h at 4°C, followed by incubation with A+ erythrocytes.

### Mouse peritoneal macrophages

C57BL/6 mice were i.p. injected or not with 1 mL of sterile 3% sodium thioglycolate (Sigma Chemical Co.). After 3 days, the animals were killed and their cells were recovered by peritoneal lavage using 5 mL of ice-cold Hank's balanced salt solution (HBSS). Cells were immediately stored in ice, washed in HBSS, suspended in RPMI-C medium [RPMI 1640 (Gibco BRL, Grand Island, NY, USA) containing 2 mM l-glutamine, 50 µM 2-mercaptoethanol, 100 units.mL^-1^ penicillin, 100 µg.mL^-1^ streptomycin (Sigma Chemical Co.), and 5% heat-inactivated fetal calf serum (FCS) (Gibco BRL)], and dispensed in 24-well cell culture plates (2×10^6^ cells.well^−1^). After a 2 h incubation at 37°C, the non-adherent cells were removed by exhaustive washing with RPMI.

Part of adherent cells were fixed with 2% paraformaldehyde at room temperature for 20 min and incubated with biotinylated Eutirucallin/streptavidin-FITC (5 µg.mL^−1^ in PBS) for 30 min. The biotinylated Eutirucallin was preincubated or not with 0.1 M D-galactose. Lectin binding was measured by flow cytometry.

Another part of adherent cell were incubated in RPMI-C, either in the presence or in the absence of Eutirucallin (1–50 µg.mL^−1^), and cultured at 37°C in a humidified 5% CO_2_ atmosphere. After 48 h, the culture supernatants were harvested by centrifugation and the measurements of cytokines and NO were performed. As positive control the macrophages were treated with LPS (1 µg.mL^−1^).

### Determination of cytokine content in the supernatants of mouse macrophage cultures

The levels of IL-12 and TNF-α were measured by capture enzyme-linked immunosorbent assay (ELISA) with antibody pairs purchased from Pharmingen (San Diego, California, USA). The ELISA procedure was performed according to the manufacturer's protocol. The cytokine concentrations were determined from a reference to a standard curve for serial twofold dilutions of murine recombinant cytokines.

### Nitrite content in the supernatant of mouse macrophage cultures

The production of NO was quantified by accumulation of nitrite in the monolayer supernatants, using the standard Griess reaction. Briefly, 50 µL of the supernatants were incubated with an equal volume of the Griess reagent (1% sulfanilamide, 0.1% naphthyl ethylenediamine dihydrochloride, 2.5% H_3_PO_4_) for 10 min. The absorbance was measured at 550 nm in the Microplate scanning spectrophotometer. The conversion of absorbance into micromolar concentrations of NO was deduced from a standard curve using a known concentration of NaNO_2_ diluted in RPMI medium.

### Neutrophil migration assay

One milliliter of lectin (1–50 µg) was injected into the peritoneal cavity of the animals. PBS was used as controls. The lectin at 10 µg was preincubated with 0.1 M of D-galactose for carbohydrate inhibition assay. Six hours later, the mice were sacrificed by cervical dislocation. The cells were immediately harvested with 5 mL PBS containing heparin (5 IU/mL). Total counts of harvested cells were performed by flow cytometry (Guava, Millipore). The results are reported as the percentage of neutrophil per mL of cavity wash.

### Preparation of peptides by mass spectrometry

The 32 kDa bands viewed by SDS-PAGE, obtained after anion exchange chromatography and affinity chromatography, were excised, fragmented with scalpel blades and transferred for microtubes (Axygen, San Francisco, CA, USA). The band fragments were washed three times with 200 µL of ammonium bicarbonate (NH4HCO3) at 25 mmol.L-1 and 50% of acetonitrile (ACN), pH 8, homogenized in vortex for 10 min; the supernatant was discarded. The samples were washed in deionized water (Milli-Q, Millipore) under the same conditions. For the reduction of polypeptide chains 25 µL (or enough to cover gel particles) of dithiothreitol at 10 mmol.L-1 in 25 mmol.L-1 de NH4HCO3, pH 8, were added to each tube, kept under stirring for 10 min. Following to that the tubes were kept at rest at 56°C for 60 min, after which the supernatant were discarded. Were added 25 µL (or enough to cover gel particles) of Iodoacetamide at 55 mmol.L-1 in 25 mmol.L-1 of NH4HCO3 for alkylation of peptides. The samples were agitated quickly, and kept at rest in the dark for 45 min. At the end of the reaction the excess alkylating agent was removed, and the gel fragments were washed again with 200 µL deionized water, dehydrated with 100 µL of ACN 100%. Partially dehydrated gel particles were subject to full Speed Vac drying (5301, Eppendorf) for 20 min without heating. The peptides were obtained by tryptic digestion after addition of 25 ng.μL-1 of trypsin “Gold” (Promega) into each tube, dissolved in 25 mmol.L-1. The supernatants obtained in tubes containing peptides were collected, and the resulting particles were washed three times. The supernatants from the washing were collected, concentrated on Speed Vac to a volume of 10 µL, and analyzed by mass spectrometry.

### Mass spectrometry

The peptides were separated by reversed-phase nano-chromatography (nanoAcquity UPLC - Waters, Milford, MA, USA) coupled with mass spectrometer nanoLC-ESI-Q-Tof MicroTM (Micromass, Waters, Milford, MA, USA). For the desalination of the samples a flow of ultrapure water (Milli-Q, Millipore) was applied at 15 µL (1 min) in Symmetry C18 pre-column. After desalting, the samples were subjected to a gradient of Acetonitrile for separation of peptides by analytical column (1.7 µm BEH300 - C18 100 µm x 100 mm) using a flow of 0.6 µL.min-1 for 50 min. The gradient was set to start with 1% of Acetonitrile for 1 min, increasing to 50% in 40 min, and finally for 85% in 5 min, kept under this condition for 2 min, and returning to 1% in 2 min, totaling 50 min. The peptides were subjected to a voltage of 3000 V for ionization in positive mode (Micromass Q-Tof MicroTM). Only peptides with types 2, 3, or 4 protonation were fragmented. The collision energy used in peptide fragmentation varied within a range of 20 to 38 eV.

### Statistical analysis

Inflammatory property of Eutirucallin results are representative of 3 independent experiments and are expressed as mean ± SD. All statistical analyses were calculated with Prism (Graph Pad Software). Comparisons between groups were made by analysis of variance, followed by Tukey's test. Statistical significance level was p<0.05.

## Results

### Protein fractions of *E. tirucalli* latex and its haemagglutinating activity

**Figure 1:**
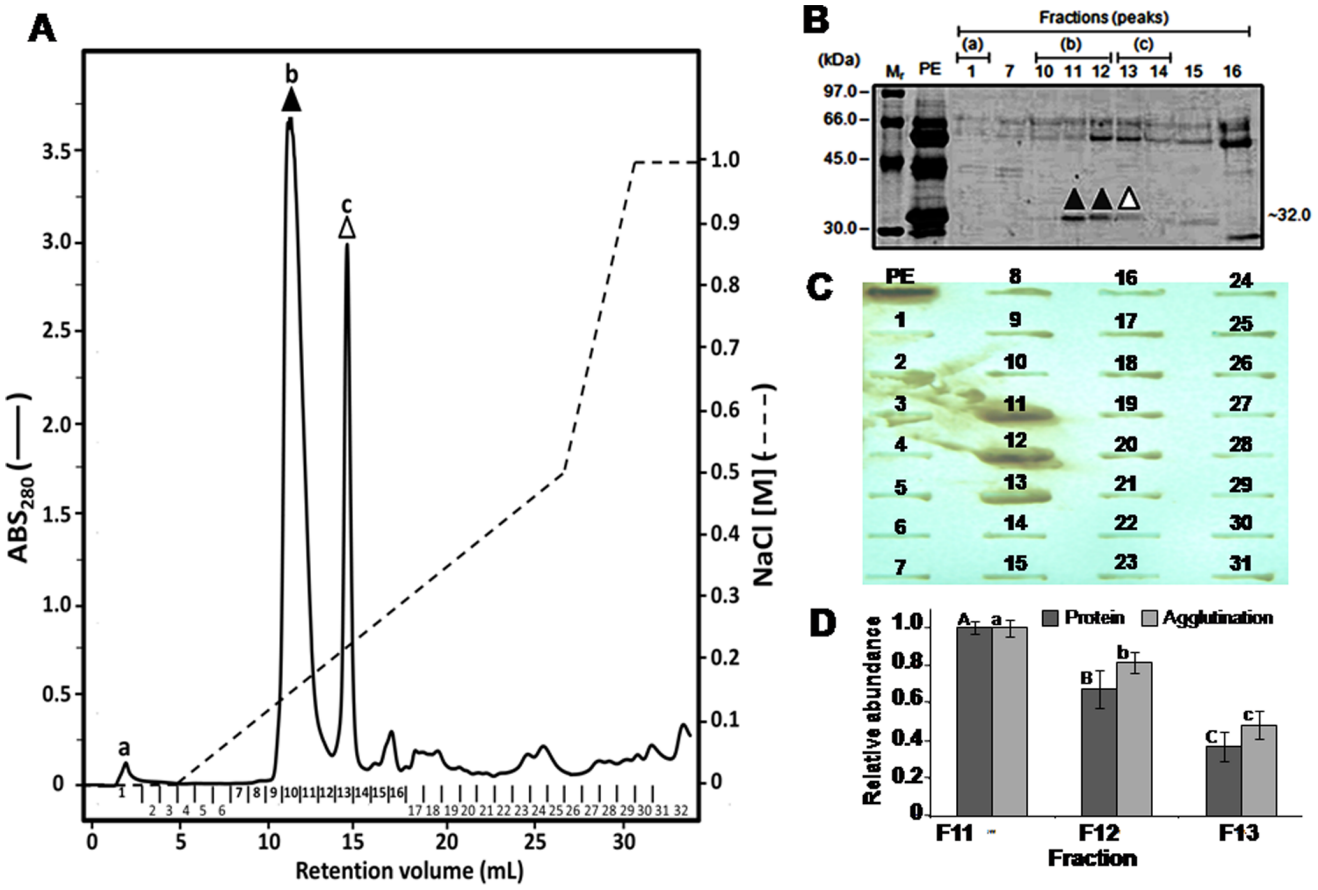
Identification of haemagglutinating fractions obtained by anion exchange chromatography. **A.** Ion exchange chromatogram of protein extract (PE) of *E. tirucalli* latex: the eluted fractions were collected (1 mL each, in a flow of 2 mL.min^−1^) and monitored spectrophotometrically in A_280nm_. All fractions obtained were tested as regards the haemagglutinating activity. Peak **a** corresponds to the non-interacted fraction; **b** and **c** represent proteins that interacted with the column. **B.** SDS-PAGE (12,5%) crude protein latex extract (PE) and fractions obtained by chromatography (1, 7, 10, 11, 12, 13, 14, 15 and 16). Closed triangles (▴) represent the haemagglutination observed after membrane washing with TBS. Mr, correspond to the molecular pattern, whose numbers on the left indicate the mass values. **C.** Determination of haemagglutinating potential of fractions by dot-blot: 200 µL aliquots of each fraction were applied on nitrocellulose membrane, and later incubated with A+ human erythrocytes (2%) for 30 min. **D.** Relative quantification of the 32KDa protein band and agglutinated eritrocites band on the nitrocellulose support by densitometric analysis using ImageMaster 2D Platinum 7.0. Vertical bars correspond to the standard deviation of the average of three replicates. Different letters indicate significant statistical difference between samples by the Tukey test (p<0,05).

Among thirty-two fractions obtained from ion exchange chromatography (Fig. 1 A), the fractions corresponding to peaks **b** and **c**, eluted in saline gradient of 180 and 250 mM, respectively, present haemagglutinating activity (Fig. 1 C). Nine out of the 32 fractions corresponding to the higher density peaks (a, b and c) and another three fractions chosen at random (7, 15 and 16), were analyzed by means of SDS-PAGE (Fig. 1 B). The fractions 11, 12 and 13 presented a polypeptide of approximately 32 kDa, absent in the remaining analyzed fractions (Fig. 1 B, bands highlighted with triangles). Furthermore these fractions promoted haemagglutination after 30 min of incubation with human erythrocytes A^+^ (Fig. 1 C). However fraction 13 haemagglutinating activitie was weaker than the observed for fractions 11 and 12. A densitometric analysis showed that the haemagglutination activity was related with the 32 KDa protein band abundance (Fig. 1B, C, D).

### Identification of the lectin in the protein extract of *E. tirucalli* latex

**Figure 2:**
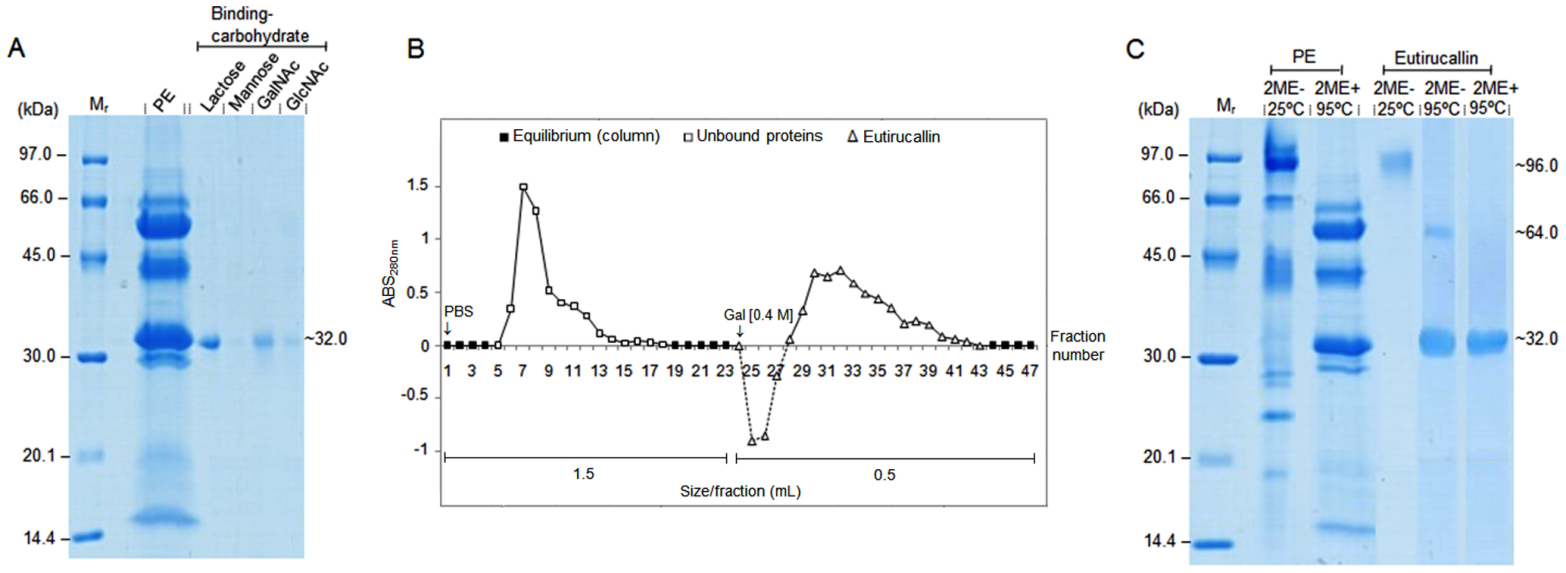
Carbohydrate specific identification and purification of Eutirucallin. **A.** Binding assay protein-carbohydrate with agarose beads adsorbed with different carbohydrates. “Beads” impregnated with different carbohydrates were incubated with the protein extract, later eluted from the resin after addition of 2-mercaptoethanol (2ME+) followed by boiling; supernatants were evaluate4d by SDS-PAGE. Lactose, mannose, N-acetyl-D-galactosamine (GalNAc) and N-acetylglucosamine (GlcNAc) correspond to the binding proteins for the corresponding sugars. **B.** Lectin purification by affinity chromatography. Approximately 2.41 mg of total protein was applied in 2 mL of α-D-Lactose column previously balanced with PBS, and eluted with galactose (0.4 M). The fractions collected during the purification were spectrophotometrically monitored (A_280nm_). **C.** Evaluation of proteins of the protein extract (PE) and Eutirucallin in bland and highly denaturating conditions. 2ME+ and 2ME- correspond, respectively, to the presence and absence of 2-mercaptoethanol. Mr, corresponds to the molecular pattern, whose numbers on the left indicate the mass values.

To confirm the presence of lectin in the *E. tirucalli* latex we performed a capture assay with different agarose-immobilized sugars (Fig. 2A). The analysis of the binding fraction with SDS-PAGE revealed a polypeptide of approximately 32 kDa able to interact with all carbohydrates used with variable affinity (Fig. 2A). Band intensity was: α-lactose > N-acetyl-D-galactosamine > N-acetyl-D-glucosamine > D-mannose. The galactosidase showed increased protein retention capacity while the others showed a discrete interaction according to the intensity of the bands in SDS-PAGE.

### Isolation and characterization of Eutirucallin from protein extract of *E. tirucalli* latex

The Eutirucallin was purified by lactose affinity chromatography (Fig. 2B) with a yield of 5.6% (Table 1). Lactose non-binding proteins correspond to fractions 6-17 and resin binding proteins were eluted with 0.4 M lactose between fractions 28–43 (Fig. 2B). The lactose affinity purified protein migrated in the SDS-PAGE with a single band of approximately 96 kDa in non-denaturing conditions (SDS only). Heating the protein at 95°C resulted in the disappearance of the 96 kDa band and the appearance of around 64 kDa (low intensity), and another around 32 kDa (high intensity). The heating combined with the presence of the 2-mercaptoethanol resulted in the presence only of the 32 kDa band (Fig. 2 C).

**Table 1:** Lectin purification.

Method obtaining	Total protein (μg)	UH^a^.(50 µL)^−1^	SHA^b^ (HU.mg^−1^)	Yield (%)
Protein extract	7,025.2	26,762.51	3,809.5	100.0
Affinity chromatography	393.41	5,359.1	13,622.1	5.6

a A unit is defined as the minimum protein concentration required, able to promote the complete agglutination of erythrocytes (title^−1^). ^b^The specific haemagglutinating activity is defined by the ratio between the UH and mg of protein used. Human erythrocytes A^+^ were used in the assay.

The crude protein extract of both *E. tirucalli* and Eutirucallin latex could agglutinate all blood types assessed (Table 2). The Eutirucallin showed specific haemagglutinating activity (SHA) of 6,808.5; for the A^+^ and O^+^ groups and 13,617.0 for the B^+^ group. Eight different carbohydrates and the EDTA were assessed to evaluate haemmaglutination inhibition (Table 3). The galactose was the only tested monosaccharide that inhibited the haemagglutinating activity from 12.5 mM (Table 3). In addition, disaccharides α-lactose (6.5 mM) and β-lactose (6.5 mM) were the most potent inhibitors.

**Table 2:** Haemagglutination assay.

	Protein extract			Eutirucallin		
Human erythrocytes (ABO)	Protein (μg)^a^	HU^b^ (50 µL)^−1^	SHA (HU.mg-1)c	Protein (μg)^a^	HUb.(50 µL)^−1^	SHA (HU.mg-1)^c^
A^+^	8.4	32	3,809.5	2.35	16	6,808.5
B^+^	8.4	32	3,809.5	2.35	32	13,617.0
O^+^	8.4	32	3,809.5	2.35	16	6,808.5

a Total amount of protein used in the assay. ^b^A unit is defined as the minimum protein concentration required, able to promote the complete agglutination of erythrocytes (title^−1^). ^c^Specific haemagglutinating activity (SHA) is defined by the ratio between the UH and mg of protein used. Human erythrocytes A^+^ were used in the assay.

**Table 3:** Haemagglutination inhibition.

Inhibitor	Conc.^a^ (mM)
*D*-dextrose	NI
*N*-acetyl-*D*-glucosamine	NI
*D*-galactose	12.5
*L*-Fucose	NI
Melibiose	25
Stachyose	50
α-lactose	6.5
β-lactose	6.5
EDTA	NI

a Minimum concentration of potential inhibitors required to promote the inhibition of the haemagglutinating activity. NI – Inhibition not observed. Human erythrocytes A^+^ were used in the assay. The sugars were tested in the concentrations of 0.78 to 50 mM. EDTA was tested in concentrations of 5, 10 and 100 mM.

**Figure 3:**
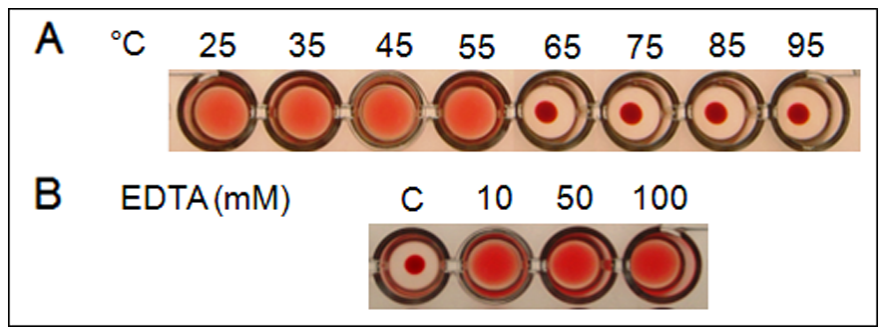
Influence of the heat treatment and EDTA on the haemagglutinating activity of the Eutirucallin. **A.** Eutirucallin (1.8 µg) was incubated for 20 min at 25, 35, 45, 55, 65, 75, 85 and 95°C. After 60 min of sedimentation at room temperature the haemagglutinating activity was evaluated. **B.** The Eutirucallin (1.8 µg) was incubated with EDTA in the concentrations of 10, 50 and 100 mM, and kept for 18 h at 4°C. Following to that the haemagglutinating activity was evaluated. Human erythrocytes A+ at 2% were used in both assays. The positive haemagglutination was indicated by the formation of a uniform coating of red blood cells in the surface of the U plates, and its absence was determined by the sedimentation of the cells in the bottom of the plates.

To assess the thermal stability of the Eutirucallin, protein aliquots were submitted to different temperatures before the haemagglutination assay (Fig. 3A). The Eutirucallin tolerated temperature below 55°C, preserving the haemagglutinating activity. The full haemagglutinating activity inhibition was observed at 65°C (Fig. 3A).

To determine whether the haemagglutinating activity of the Eutirucallin depends on Ca^2+^ ions, the EDTA was tested in reaction media in the concentrations of 10, 50 and 100 mM (Fig. 3B and Table 3). The EDTA did not inhibit the haemagglutinating activity after 18 h of incubation with the Eutirucallin (Fig. 3B).

### Immunostimulatory property of Eutirucallin

**Figure 4:**
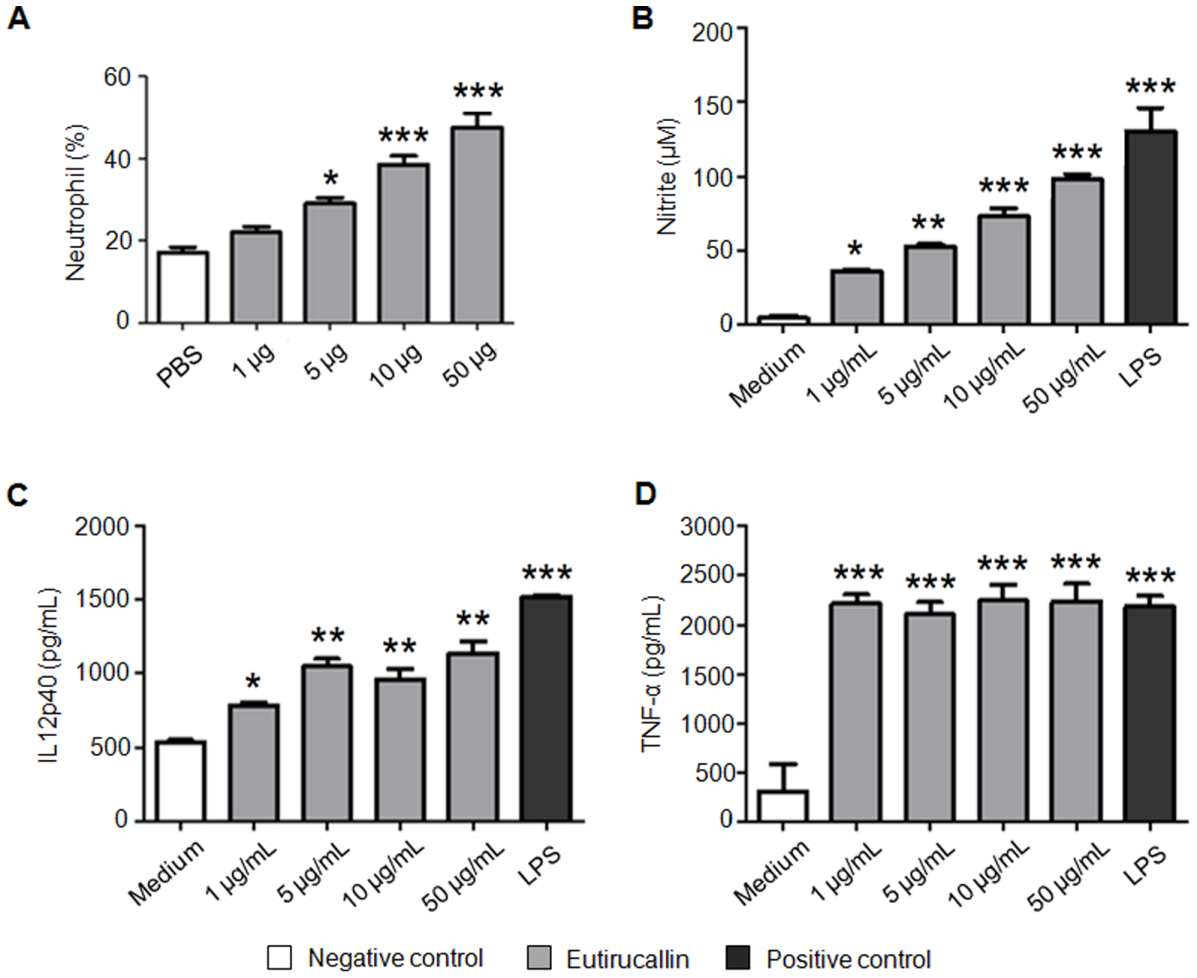
Inflammatory property of Eutirucallin. **A.** Eutirucallin (1–50 µg) was injected into the peritoneal cavity of the animals. Six hours later the total counts of harvested cells were performed by flow cytometry. The results are reported as the percentage of neutrophil per ml of cavity wash. PBS was used as controls. Adherent cells from the peritoneal cavities of C57BL/6 mice were stimulated in vitro for 48 h with Eutirucallin (1–50 µg/mL), medium (negative control) or LPS (positive control). The culture supernatants were assayed for NO (**B**), IL12p40 (**C**) and TNF-α (**D**) levels. *p<0.05,**p<0.01, and ***p<0.001 comparing with medium.

The inflammatory property of lectin was evaluated using neutrophil chemotactic assay and macrophage pro-inflammatory products measurement. To examine the dose-dependent activity of lectin, we injected, intraperitoneally, 1–50 µg of Eutirucallin in mice. Exudates cell counting carried out 6 h after the injection showed that preparations induced neutrophil migration into the peritoneal cavity in a dosedependent manner (Fig. 4A). The carbohydrate inhibition assay was performed in migration assay and none inhibition was observed. In presence of 10 µg of Eutirucallin, 47 ± 2% of neutrophils were observed into the peritoneal cavity whereas in presence of 10 µg of Eutirucallin preincubated with 0.1 M of D-galactose group 48 ±6% of neutrophils were observed.

The effect of lectin on the production of pro-inflammatory IL12 and TNF-α cytokines and nitric oxide (NO) was determined in peritoneal macrophages (Fig. 4 B,C,D).

A dose-response was observed by NO detection. The levels of IL-12 and TNF-α was raised at all doses. Indeed, it was observed the interaction of biotinylated Eutirucallin with 99% of peritoneal macrophages and the mean fluorescence intensity (MFI) was 82 ± 2. None inhibition was observed by preincubation of lectin with 0.1 M of D-galactose (99% cell binding and MFI = 84 ± 1).

### Similarity of Eutirucallin with type 2 ribosome-inactivating protein

**Figure 5:**
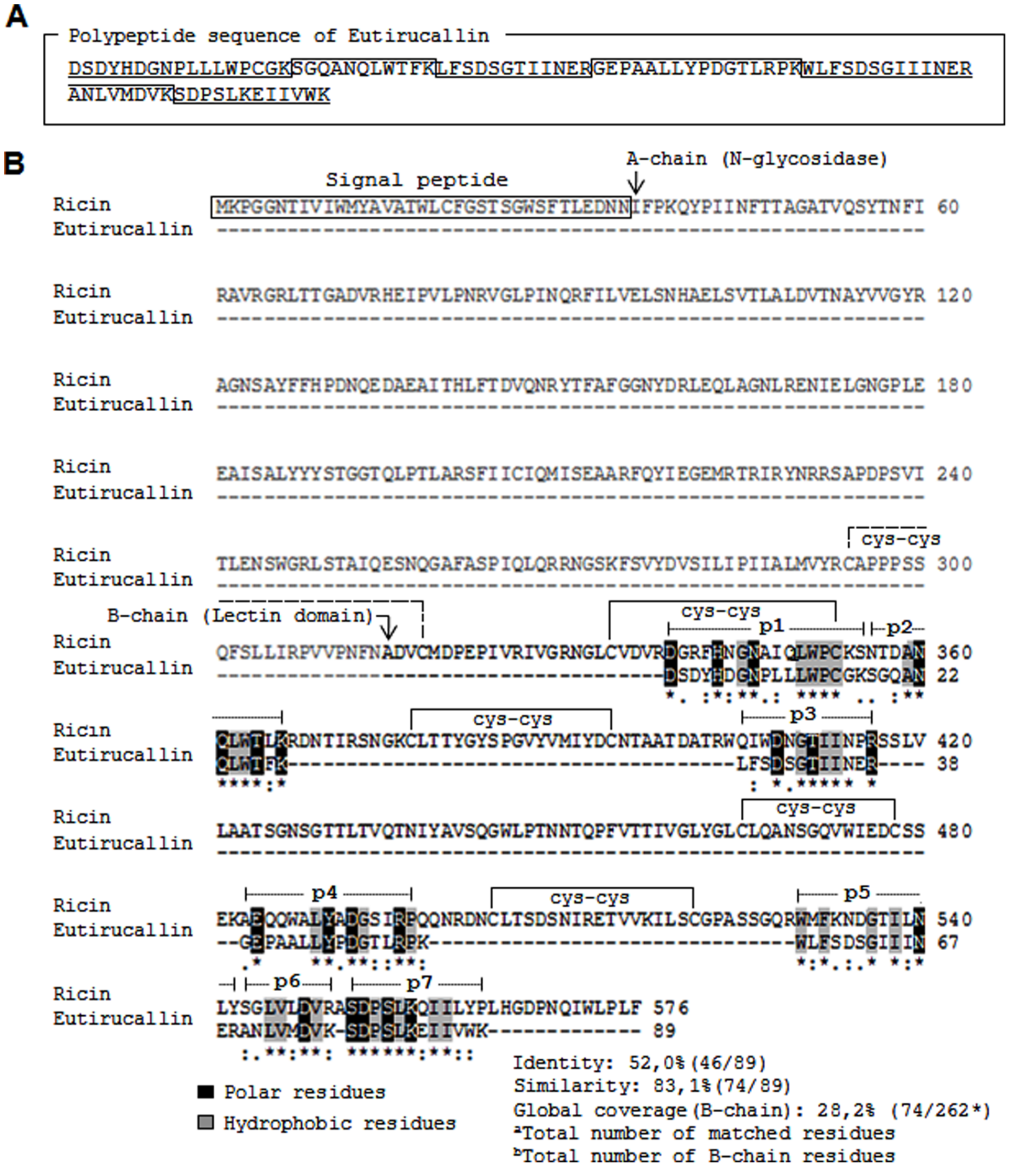
Construction of the polypeptide sequence of Eutirucallin. A. Cluster assembled from of peptides obtained by ms/ms. B. The order of peptides in the cluster was established by alignment with ricin. The letters “single letter code” correspond to the amino acids residues. (*) Represent identical residues; (:) conserved residues; and (.) semi-conserved replacements. The arrow (↓) corresponds to the initial residues of the A- (N-glycosidase) and B- (lectin domain) chains, respectively. The signal peptide is indicated in the sequence initial segment. The residues (cys-cys) correspond to the intramolecular and intermolecular disulfide bindings, indicated by the full and dashed lines, respectively.

**Figure 6:**
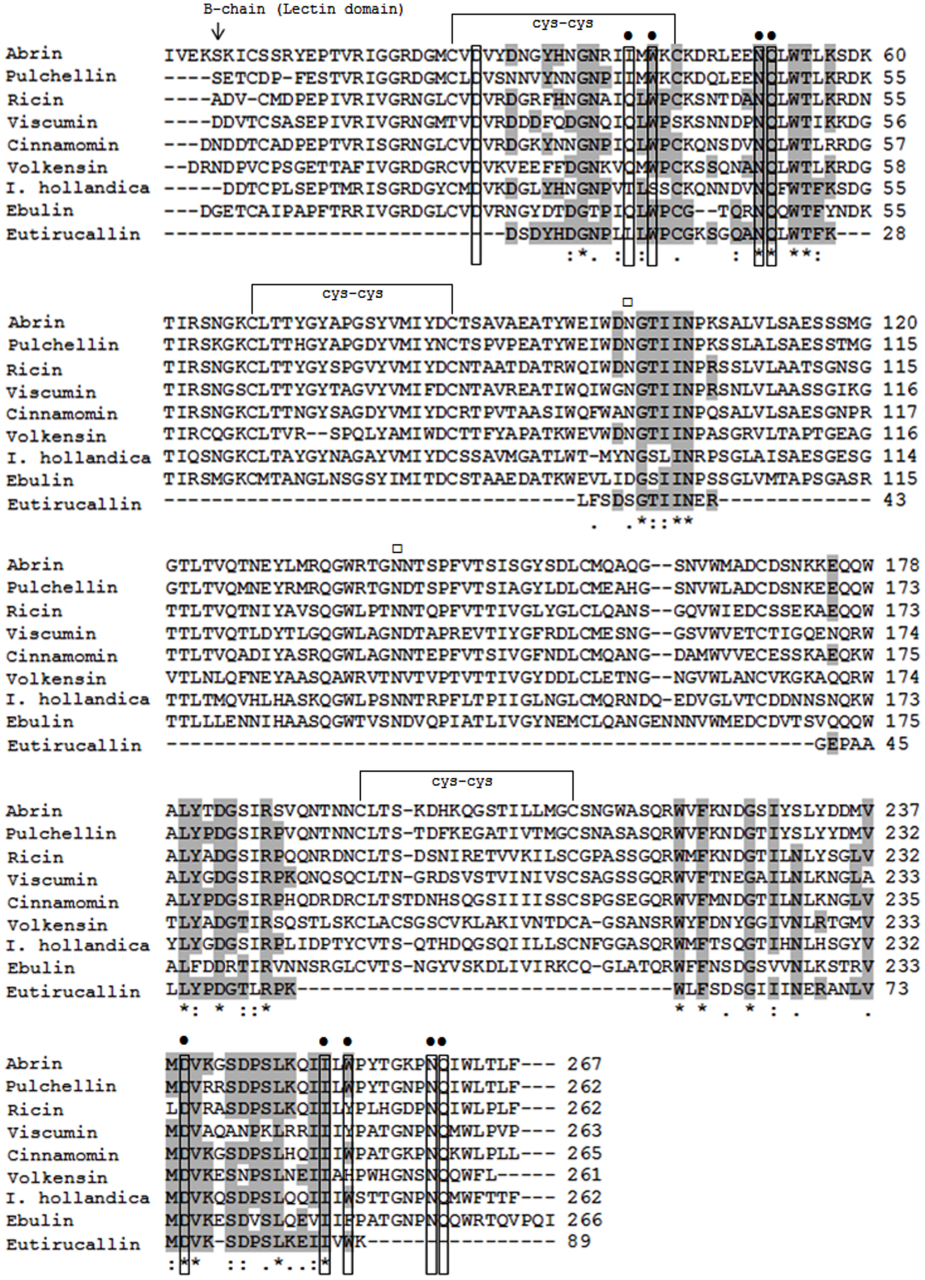
Alignment of partial sequence of Eutirucallin with type 2 ribosome-inactivating protein. All identity and similarity analyses were performed with the tool clustalw2. The sequences used were: Ricin (GenBank: XP_002534649.1), Abrin a (PDB: 2AAI_B), Viscumin (PDB: 2RG9_B), Ebulin (PDB: 1HWM_B), Pulchellin (GenBank: AAM19073.1), Cinnamomin (GenBank: AAK82460.1), Volkensin (GenBank: CAD61022.1) and type 2 RIP of *Iris hollandica* (GenBank: AAL55093.1). The letters “single letter code” correspond to amino acids residues. (*) Represent identical residues; ( ) residues identical for at least one of the sequences; (:) conserved residues; and (.) semi-conserved replacements. The arrow (↓) corresponds to the initial residue of B-chain (lectin domain). The residues involved in the glycidic recognition are marked with closed circles (•); the probable glycosylation points are represented by open squares (□).

The 32 kDa band corresponding to the Eutirucallin purified by lactose affinity (Fig. 2C) was submitted to tryptic digestion and mass spectrometry in NanoUPLC-ESI Q-Tof, to establish the protein's identity. Nine tryptic peptides separated by the NanoUPLC were fragmented with manual selection and the sequences were generated by ms/ms (Table 4). The peptide sequence was analyzed in comparison with the NCBI databank. All peptides showed variable identity with type 2 ribosome-inactivating protein (RIPs) of different species (Table 4). The alignment developed with peptides individually evaluated showed variable identity from 30.8 to 69.7%, and similarity from 69.2 to 100% (data not shown) with Ricin (type 2 RIP of *Ricinus communis*). The peptide sequence was clustered based on the position of each peptide in the alignment with the Ricin (Fig. 5A). Seven of the nine peptides of the cluster showed 83.1% of coverage with the Ricin sequence, all of which in the protein's carboxy-terminal region (Fig. 5B). The partial sequence of the Eutirucallin was compared to the lectin domain regions (B-chain) of eight type 2 RIPs (Ricin, Abrin, Viscumin, Ebulin, Pulchellin, Cinnamomin, Volkensin and type 2 RIP of *Iris hollandica*) (Fig. 6) and presented identities of 41.5–52% and similarities of 67.4–83.1%. The higher similarity (83.1%) occurred with Ricin and Abrin a (Table 5).

**Table 4:** Tryptic peptides sequenced by ms/ms from the 32 kDa band of the Eutirucallin and proteins identified from the search performed at the NCBI's databank.

Peptide description			Top-hit protein^b^		
Sequence^a^	Mass/Charge ratio (m/z)	Charge	Access Number	Description	Specie
ANLVMDVK	445.22	2+	AAF37218.1	Ribosome-inactivating protein	*Polygonatum multiflorum*
SGQANQLWTFK	640.26	2+	CAD61022.1	Volkensin	*Adenia volkensii*
SDPSLKEIIVWK	707.87	2+	XP_002532189.1	Ricin-agglutinin family protein	*Ricinus communis*
DSDYHDGNPLLLWPCGK	965.37	2+	AAR25549.1	Lectin precursor	*Viscum album*
GEPAALLYPDGTLRPK	849.37	2+	2VLC_A	Chain A, Natural Cinnamomin	*Cinnamomum Camphora*
WLFSDSGIIINER	769.35	2+	XP_002534513.1	Conserved hypothetical protein	*Ricinus communis*
LSSSGPDGYGCVDVK	742.30	2+	AAL55093.1	ribosome-inactivating protein IRAr	*Iris hollandica*
LFSDSGTIINER	676.32	2+	ADG29117.1	Preproricin	*Ricinus communis*
SDDYHDGNPIILWQEK	965.37	2+	PDB: 2Q3N_B	Chain B, agglutinin (Apa-I)	*Abrus precatorius*

a Peptide sequence obtained by ms/ms in NanoUPLC-ESI Q-Tof. ^b^The search for biological sequences related was performed at the protein databank of the NCBI (GenBank) non redundant.

**Table 5:** Comparative analysis of similarity/identity of the Eutirucallin sequence with eight B-chains of type 2 ribosome- inactivating protein.

	B-chain^b^								
Similarity^a^/Identity^a^	Eutirucallin	Ricin	Abrin	Viscumin	Ebulin	Pulchellin	Cinnamomin	Volkensin	*I. hollandica*
Eutirucallin	----	83.1	83.1	77.5	67.4	77.5	82.0	77.5	77.5
Ricin	52.0	----	90.0	93.3	79.5	88.8	96.7	76.7	85.4
Abrin	49.4	67.7	----	93.0	76.1	95.0	92.2	78.8	79.5
Viscumin	44.9	65.5	50.0	----	78.6	85.5	92.2	90.0	83.2
Ebulin	41.5	47.7	46.6	40.9	----	74.0	81.8	76.1	75.0
Pulchellin	48.3	68.5	86.6	51.1	44.4	----	82.2	82.2	84.3
Cinnamomin	52.8	73.3	63.3	64.4	50.0	65.5	----	85.5	83.1
Volkensin	49.4	47.7	55.5	54.4	48.9	52.2	55.5	----	77.5
*I. hollandica*	46.0	57.3	51.1	52.0	44.3	50.5	62.9	44.9	----

a The identity amounts are presented below the axis (----) and represents the percentage of identical amino acids residues. Similarity amounts are presented above the diagonal axis. ^b^The amounts were obtained by considering only the paired regions of the polypeptide sequence of the Eutirucallin. All analyses were made with the tool Clustalw2.

## Discussion

In this study we have identified and isolated a lectin in the aqueous extract of *E. tirucalli* latex, which was named Eutirucallin. Initially, the haemagglutinating activity of anion exchange chromatography fractions was tested. It is considered an important initial step for the lectin investigation in plant extracts [9], [10]. By means of this approach we identified an agglutinin with molecular mass of approximately 32 kDa. The main advantages of this semi-quantitative technique are the speed in the implementation and non-dependence on desalination of the fractions by dialysis and protein concentration, which are normally required in the lectin research in extracts. The method used also enables the direct viewing and photo-documentation of the result for a large number of fractions simultaneously. After that, we carried out protein-carbohydrate-agarose interaction assays using 32 kDa polypeptide. We noticed predilection for the resins adsorbed by α-lactose residues, followed by *N*-acetyl-D-galactosamine, proposing the first indication that this is a binding lectin of galactose/*N*-acetylgalactosamine (Gal/GalNAc), with counterparts described for several members of the Euphorbiaceae family [11], [12]. Although many lectins present monosaccharide specificity, usually there is more avidity to more complex sugars [13], which may explain the higher interaction capacity of the Eutirucallin with the lactose disaccharide (4-O-β-D-galactopyranosyl-D-glucose). This suggests the involvement of the globular domain of carbohydrate recognition (DRC) in recognizing the β-*D*-galactosidase mediated by intramolecular (van de Waals) and intermolecular interactions (hydrogen bridges and hydrophobic interactions) [13], [14]. Because of this and due to the higher protein retention capacity observed for the α-lactose resin, the α-lactose was used in Eutirucallin purification assays by affinity chromatography (yield of 5.6%).

Analyses carried out with different denaturing treatments by SDS-PAGE showed that Eutirucallin is an oligomer of approximately 96 kDa, constituted by three 32 kDa polypeptides, and at least two of them are bound by disulfide bridges. In the same way, RIPs are shown as a heterodimeric molecule with approximate molecular weight of 30 kDa per unit, where each protomer presents two functional polypeptides (A- and B-chains) disposed in tandem and connected by disulfide bridges [15], [16]. Our findings propose that Eutirucallin is a trimeric molecule due to the existence of an additional polypeptide with molecular weight (∼32 kDa) guaranteeing a structural uniqueness in comparison to other RIPs.

The galactosidase specificity was confirmed after the inhibition test with monosaccharides, showing that galactose could completely inhibit the haemagglutinating activity (B^+^) in the concentration 12.5 mM. Additional agglutination and inhibition assays of the haemagglutinating activity were performed to investigate the molecule's selectivity by different blood types, and also its glycidic specificity. Both the protein extract and the Eutirucallin could promote agglutination of all erythrocytes evaluated (A^+^, B^+^ and O^+^), showing that the lectin does not present group-specific selectivity for human red blood cells, although the molecule has presented a slight preference for erythrocytes B^+^. This behavior may be explained by the fact that all ABO groups are composed by glycidic antigens containing galactoside moieties (α-*N*-acetyl-D-glucosaminide, α-D-galactose and α-L-fucose, characterizing the A, B and O groups, respectively) [17]. This property is usually described for several members of the Gal/GalNAc binding lectins [1], [18], [19].

The Eutirucallin's carbohydrate recognition domain of Eutirucallin does not depend on metal coordination by Ca^2+^ ions for the agglutinating activity, as the EDTA could not inhibit the Eutirucallin agglutination in concentration of up to 0.1 M (Fig. 3 and Table 3). Fine structural studies show that divalent metal ions such as Ca^2+^, Mn^2+^ or Mg^2+^, are important for the folding conformation of several lectins and may be directly or indirectly involved in the binding with the saccharide [20], [21], [22].

The thermal stability test with the lectin showed complete inhibiton of haemagglutinating activity in temperature equal or higher than 65°C. Data prove that the B-chain in RIPs is responsible for this property. An assay carried out with cinnamomin [15] showed that temperatures higher than 45°C is responsable to promotes conformational changes in B-chain lead to loss of *N*-gycosidase activity in A-chain.

Several lectins obtained from vegetal sources have been reported for promoting biological responses such as the recruitment of neutrophils and activation of macrophages with production of TNF-α and IL-12, important for the development of the inflammatory process and modulation of the immune response that culminates with the resolution of infections [23], [24], [25]. The Eutirucallin lectin has immune-stimulator potential, promoting the recruitment of neutrophils in vitro and inducing the production of TNF-α, IL12 and NO by murine macrophages (Fig. 4). However, neither the neutrophil migration nor the lectin-macrophage interaction were inhibited by the presence of the galactose. Thus, two assumptions are proposed: *(i)* there are a complex glycans in the macrophages, more strongly recognized by lectin than galactose; *(ii)* or the biological response observed is not mediated by carbohydrate recognition domain.

Studies prove that the RIPs present great avidity for galactosyl moieties of complex glycans such as glycoproteins or glycolipids present on the surface of eukaryotic cells [26], [27]. On the other hand, Yamazaki and cols [28] assessed the inducing capacity for the production of cytokines by different RNA *N*-glycosidase, among which RIPs such as ricin and modeccin, and observed the inducing capacity of cytokines. In view of this, in accordance with our results, we suggest that the immunostimulatory activity of the molecule may be related to the A-chain (with *N*-glucosidase activity), and not the B-chain which represents the lectin domain.

We have identified nine trypsin digested peptides of Eutirucallin, which showed similarity with type 2 ribosome-inactivating proteins with species that are not taxonomically related. The lack of a genome sequence for *E. tirucalli* confirmed after searches in the GenBank (NCBI) made us adopt a new approach to prove that Eutirucallin belong to the type 2 ribosome-inactivating protein family (type 2 RIPs). From of three peptides, three sequences showed similarity to ricin as “top-hit”, which led us to use it as reference for the clustering of the Eutirucallin peptides. This cluster presented coverage of 28.2% (74/262) for the global ricin chain, and 83.1% (54/89) for the lectin domain (B-chain). The alignment of the partial sequence of Eutirucallin with eight sequences of type 2 RIPs showed similarities of 67.4 to 96.7% between the RIPs evaluated. The majority of conserved amino acid residues for the eight lectins are also preserved in the partial sequence of the Eutirucallin. The analyses showed an incredible conservation of residues present in carbohydrate-binding sites. Conserved segments residue-rich of hydrophobic amino acids interspersed by small polar extensions, which increase the stability of the binding in the gap by means of hydrophobic, van der waals and electrostatic interactions, guarantying a high avidity with binding sugar, are present in these sites [13]. Even lectins with low sequence identity of amino acids may assume similar conformational structures [29]. Evolutionary aspects of lectin domain conservation for carbohydrates recognition (CRD) are related to the evolution convergence process [29], [30]. In view of this, discrete changes observed in the protein sequence do not condition significant conformational changes, preserving the specificity of the glycidic molecule. Similarly, these differences observed for the CRD between different RIPs and the Eutirucallin are fully acceptable. Therefore, the existing conformational conservation may be related to similar functional needs of molecules granting, in this case, the Eutirucallin's specificity by galactosidase (Gal/GalNAc).

Although biological assays of specific inhibition of ribosomal activity are required, all data to a type 2 ribosomes inactivating protein: (*i*) showed affinity with Gal/GalNAc; (*ii*) it has at least two polypeptides (32.000 Da) composing the lectin domain connected by disulfide bindings; (*iii*) it is a potent haemagglutinin able to agglutinate ABO erythrocytes; (*iv*) and the toxicity attached to the latex of *E. tirucalli*.

## Supporting Information

Figure S1 **Eutirucallin binding to macrophages.** Flow cytometry histogram overlays show biotinylated Eutirucallin/streptavidin-FITC binding to macrophages in the absence (filled dark gray) or presence of 0.1 M D-galactose (empty black). The fluorescence background (macrophage incubated with streptabidin-FITC) as shown in filled light gray.
(DOCX)
