# Supplementary figures and images for: Eutirucallin, a RIP-2 Type Lectin from the Latex of Euphorbia tirucalli L. Presents Proinflammatory Properties
Source: PLoS One. 2014 Feb 18;9(2):e88422. doi: 10.1371/journal.pone.0088422 (PMC3928152; doi:10.1371/journal.pone.0088422)

**Figure S1**


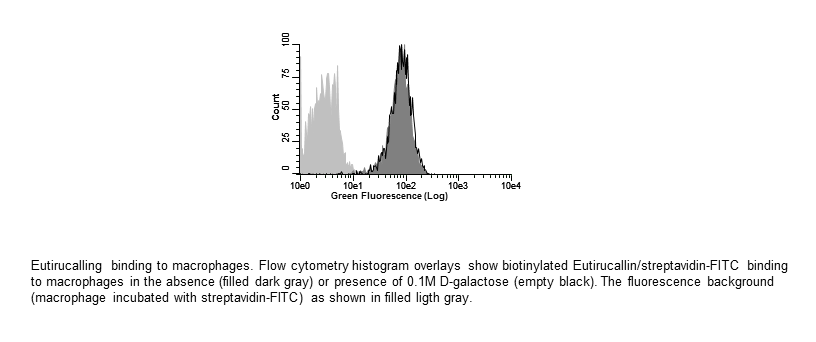

Supplement: Figure S1 — Eutirucallin binding to macrophages. Flow cytometry histogram overlays show biotinylated Eutirucallin/streptavidin-FITC binding to macrophages in the absence (filled dark gray) or presence of 0.1 M D-galactose (empty black). The fluorescence background (macrophage incubated with streptabidin-FITC) as shown in filled light gray. (DOCX) [file pone.0088422.s001.docx]
